# Supplementary material for: Trans-Presentation of IL-15 by IL15Rα Attenuates Tumor Immune Surveillance and Is Dispensable for IL-15-Dependent Tumor Growth Control
Source: Cancers (Basel). 2026 Aug 4;18(15):2499. doi: 10.3390/cancers18152499 (PMC13465580; doi:10.3390/cancers18152499)
Supplement: Supplementary file 1 [file cancers-18-02499-s001.zip › Supplementary Figures and Tables.pdf]

Supplementary Materials

Table S1. List of primers used for RT-qPCR.

Gene name	Gene ID	Sense primer	Anti-sense primer	Amplicon Size (bp)
Cxcl9	NM_008599.4	AGTCCGCTGTTCTTTTCCTC	TGAGGTCTTTGAGGGATTTGTAG	141
Cxcl10	NM_021274.2	CCAAGTGCTGCCGTCATTTTC	GGCTCGCAGGGATGATTTCAA	157
Cd8a	NM_001081110.2	CATCACTCTCATCTGCTACCAC	TTTTCTCTGAAGGTCTGGGC	98
Cd4	NM_013488.3	GTTCGGCATGACACTCTCAG	CCTTCTCTGCCTTCCACATC	146
Klrb1c (NK1.1)	NM_001159904.2	TGGCACAACTTTCAATTCTGATG	GGTTTAGTTCCTTTTGGCAGATC	140
Cd274 (PD-L1)	NM_021893.3	CTCATTGTAGTGTCCACGGTC	ACGATCAGAGGGTTCAACAC	149
Ifng	NM_008337.4	CCTAGCTCTGAGACAATGAACG	TTCCACATCTATGCCACTTGAG	150
Ifnb1	NM_010510.2	TGTCCTCAACTGCTCTCCAC	CTGAAGATCTCTGCTCGGACC	112
Klrd1	NM_010654.5	TCAACACCTTCTCCAACCAC	CTGATGCCCAACCCACTT	84
Klrc2	NM_010653.5	AATCTCTTTCACTGGTCTCATGG	GCAAATTCATCTAAAGGGAGCC	262
Klrk1	NM_033078.4	GCTGGTTAAGTCCTATCACTGG	TTGAGCCATAGACAGCACAG	143
36B4 (Rplp0)	NM_007475.5	TCTGGAGGGTGTCCGCAAC	CTTGACCTTTTCAGTAAGTGG	154

Table S2. List of antibodies used for flow cytometry on a Cytoflex 30 flow cytometer.

Antibody	Fluorophore	Clone	Company	Cat#
Flexible Viability Dye	eFluor 780	-	eBiosciences	65-0865-18
CD45	Brilliant Violet 605	30-F11	Biolegend	103140
CD19	PE	6D5	Biolegend	115508
NK1.1	APC-Cy7	PK136	Biolegend	108724
TCRb	PE/Dazzle594	H57-597	Biolegend	109240
CD8a	eFluor450	53-6.7	eBiosciences	48-0081-82
CD4	AlexaFluor700	GK1.5	eBiosciences	50-168-51
CD62L	APC	MEL-14	eBiosciences	17-0621-83
CD44	FITC	IM7	Biolegend	103006
CD69	PECy7	H1.2F3	Biolegend	104512
CD279 (PD-1)	BV785	29F.1A12	Biolegend	135225
TCRgd	PerCP	GL3	Biolegend	118117
CD45	Brilliant Violet 510	30-F11	Biolegend	742863
Ly6G	PerCP	1A8	Biolegend	127654
CD11c	Alexa Fluor 700	N418	eBiosciences	56-0114-82
CD11b	eFluor450	M1/70	eBiosciences	48-0112-82
MHC II	PECy7	M5/114.15.2	Biolegend	107630
Ly6C	Alexa Fluor 647	HK1.4	Biolegend	128016
F4/80	Brilliant Violet 605	BM8	Biolegend	123133
CD122	FITC	TM-B1	eBiosciences	11-1222-82
CCR2	PE	SA203G11	Biolegend	150610
CX3CR1	PE/Dazzle 594	SA011F11	Biolegend	149013

Table S3. List of antibodies used for flow cytometry on a BD FACSDiscover™ S8 cell sorter.

Antibody	Fluorophore	Clone	Company	Cat#
CD197 (CCR7)	BV605	4B12	BD Biosciences	740431
CX3CR1	BB700	Z8-50 (Z8-50.23)	BD Biosciences	567812
CD185 (CXCR5)	RB613	2G8	BD Biosciences	759651
CD194 (CCR4)	PE-Cy7	2G12	BioLegend	131214
CD183 (CXCR3)	PE-Fire640	S18001A	BioLegend	155922(BLG)
CD197 (CCR7)	BV605	4B12	BD Biosciences	740431
CD69	BUV805	30-F11	BD Biosciences	741927
Ly6G	BUV737	1A8	BD Biosciences	568346
Ly49A	BUV661	A1	BD Biosciences	749810
CD27	BUV615	LG.3A10	BD Biosciences	751529
NK1.1	BUV563	PK136	BD Biosciences	741233
CD45	BUV496	145-2C11	BD Biosciences	569673
Live/Dead (FVS440 UV)	FVS440UV	-	BD Biosciences	566332
XCR1	Spark UV 387	ZET	BioLegend	148236(BLG)
CD62L	BUV395	MEL-14	BD Biosciences	569400
MHC-I	BV786	AF6-88.5	BD Biosciences	742863
CD279 (PD-1)	BV750	29F.1A12	BD Biosciences	568635
CD107a	BV711	1D4B	BD Biosciences	564348
CD64 A/B	BV650	X54-5/7.1	BD Biosciences	740622
CD11c	BV510	HL3	BD Biosciences	562949
CD44	BV480	IM7	BD Biosciences	566116
CD4	RV828	GK1.5	BD Biosciences	5726748
TIGIT	BV421	1G9	BD Biosciences	565270
CD152 (CTLA-4)	PE	BNI3	BD Biosciences	553720
CD11b	PerCP-Fire806	M1/70	BioLegend	101294
Singlec-H	RB780	440c	BD Biosciences	755362
CD103	RB744	M290	BD Biosciences	758108
TCRgd	RB705	GL3	BD Biosciences	757547
CRIg (VSIG4)	RB670	17C9	BD Biosciences	771636
CD49a	RB545	Ha31/8	BD Biosciences	756784
CD25	BB515	PC61	BD Biosciences	564424
I-A/I-E (HLA-DR)	PE-Fire810	M5/114.15.2	BioLegend	107667
CD206	PE-Fire700	C068C2	BioLegend	141742(BLG)
IgD	PE-Cy5	11-26c-2a	BD Biosciences	624350
TIM-4	RY610	21H12	BD Biosciences	758786
CD8a	RY743	53-6.7	BD Biosciences	572203
CD19	RY586	1D3	BD Biosciences	753680
CD20	APC-Fire810	SA275A11	BioLegend	150438
Ly-6C	APC-Cy7	AL-21	BD Biosciences	560596
CD274 (PD-L1)	R718	10F.9G2	BD Biosciences	568585
CD3e	APC	H1.2F3	BD Biosciences	565643
F4/80	AF647	T45-2342	BD Biosciences	565853

Table S4. Phenotype markers defining immune cell subsets.

Figure	Cell types	Phenotype
Figure 9B	NK cells	$CD45^{+}Ly6G^{-}CD19^{-}CD20^{-}CD3^{-}CD49a^{-}NK1.1^{+}$
Figure 9C	ILC1-like cells	$CD45^{+}Ly6G^{-}CD19^{-}CD20^{-}CD3^{-}CD49a^{+}NK1.1^{+}$
Figure 9D	NKT cells	$CD45^{+}Ly6G^{-}CD19^{-}CD20^{-}CD3^{+/-}TCRgd^{-}CD4^{+}NK1.1^{-}$
Figure 9E	TCRγδ T cells	$CD45^{+}Ly6G^{-}CD19^{-}CD20^{-}CD3^{+/-}TCRgd^{+}$
Figure 9F	Total $CD4^{+}$ T cells	$CD45^{+}Ly6G^{-}CD19^{-}CD20^{-}CD3^{-/+}TCRgd^{-}CD8^{-}CD4^{+}NK1.1^{-}$
Figure 9G	$CD4^{+}$ naïve T cells	$CD45^{+}Ly6G^{-}CD19^{-}CD20^{-}CD3^{-/+}TCRgd^{-}CD8^{-}CD4^{+}NK1.1^{-}CD25^{-}CD62L^{+}CD44^{-}$
Figure 9H	$CD4^{+}$ Cm T cells	$CD45^{+}Ly6G^{-}CD19^{-}CD20^{-}CD3^{-/+}TCRgd^{-}CD8^{-}CD4^{+}CD62L^{+}CD44^{+}$
Figure 9I	$CD4^{+}$ Eff T cells	$CD45^{+}Ly6G^{-}CD19^{-}CD20^{-}CD3^{-/+}TCRgd^{-}CD8^{-}CD4^{+}CD62L^{-}CD44^{+}$
Figure 9J	$CD4^{+}$ Treg-enriched	$CD45^{+}Ly6G^{-}CD19^{-}CD20^{-}CD3^{-/+}TCRgd^{-}CD8^{-}CD4^{+}NK1.1^{-}CD25^{+}CD103^{+}$
Figure 9K	Total $CD8^{+}$ T cells	$CD45^{+}Ly6G^{-}CD19^{-}CD20^{-}CD3^{-/+}TCRgd^{-}CD8^{+}CD4^{-}$
Figure 9L	$CD8^{+}$ naïve T cells	$CD45^{+}Ly6G^{-}CD19^{-}CD20^{-}CD3^{-/+}TCRgd^{-}CD8^{+}CD4^{-}CD62L^{+}CD44^{-}$
Figure 9M	$CD8^{+}$ Cm T cells	$CD45^{+}Ly6G^{-}CD19^{-}CD20^{-}CD3^{-/+}TCRgd^{-}CD8^{+}CD4^{-}CD62L^{+}CD44^{+}$
Figure 9N	$CD8^{+}$ Eff T cells	$CD45^{+}Ly6G^{-}CD19^{-}CD20^{-}CD3^{-/+}TCRgd^{-}CD8^{+}CD4^{-}CD62L^{-}CD44^{+}$
Figure 9O	$TIGIT^{+}$ Eff $CD8^{+}$ T cells	$CD45^{+}Ly6G^{-}CD19^{-}CD20^{-}CD3^{-/+}TCRgd^{-}CD8^{+}CD4^{-}CD62L^{-}CD44^{+}TIGIT^{+}$
Figure 9P	$CD8^{+}PD\text{-}1^{+}CX3CR1^{+}TIGIT^{+}$ Eff T cells	$CD45^{+}Ly6G^{-}CD19^{-}CD20^{-}CD3^{-/+}TCRgd^{-}CD8^{+}CD4^{-}CD62L^{-}CD44^{+}$
Figure 10A	$Ly6G^{+}$ neutrophils	$CD45^{+}Ly6G^{+}Ly6C^{lo}CD11b^{+}$
Figure 10B	TAMs	$CD45^{+}Ly6G^{-}CD19^{-}CD20^{-}CD3^{+/-}TCRgd^{-}CD4^{-}CD8^{-}NK1.1^{-}CD11b^{+}CD11c^{-}F4/80^{+}CD206^{+}$
Figure 10C	MODCs	$CD45^{+}Ly6G^{-}CD19^{-}CD20^{-}CD3^{+/-}TCRgd^{-}CD4^{-}CD8^{-}NK1.1^{-}CD11b^{+}CD11c^{+}$
Figure 10D	$CD11c^{+}$DCs	$CD45^{+}Ly6G^{-}CD19^{-}CD20^{-}CD3^{+/-}TCRgd^{-}CD4^{-}CD8^{-}NK1.1^{-}CD11b^{-}CD11c^{+}$
Figure 10E	Monocytes	$CD45^{+}Ly6G^{-}CD19^{-}CD20^{-}CD3^{+/-}TCRgd^{-}CD4^{-}CD8^{-}NK1.1^{-}CD11b^{+}CD11c^{-}$
Figure 10F	Monocytes $F4/80^{-}CD206^{-}Ly6C^{+}$	$CD45^{+}Ly6G^{-}CD19^{-}CD20^{-}CD3^{+/-}TCRgd^{-}CD4^{-}CD8^{-}NK1.1^{-}CD11b^{+}CD11c^{-}F4/80^{-}CD206^{-}Ly6C^{+}$
Figure 10G	Monocytes $F4/80^{+}CD206^{-}$	$CD45^{+}Ly6G^{-}CD19^{-}CD20^{-}CD3^{+/-}TCRgd^{-}CD4^{-}CD8^{-}NK1.1^{-}CD11b^{+}CD11c^{-}F4/80^{+}CD206^{-}$
Figure 10H	Monocytes $F4/80^{+}CD206^{-}Ly6C^{+}$	$CD45^{+}Ly6G^{-}CD19^{-}CD20^{-}CD3^{+/-}TCRgd^{-}CD4^{-}CD8^{-}NK1.1^{-}CD11b^{+}CD11c^{-}F4/80^{+}CD206^{-}Ly6C^{+}$
Figure 10I	$CD11c^{-}CD11b^{-}$ immature monocytes	$CD45^{+}Ly6G^{-}CD19^{-}CD20^{-}CD3^{+/-}TCRgd^{-}CD4^{-}CD8^{-}NK1.1^{-}CD11b^{-}CD11c^{-}$
Figure 10J	$CD11c^{-}CD11b^{-}Ly6C^{+}$	$CD45^{+}Ly6G^{-}CD19^{-}CD20^{-}CD3^{+/-}TCRgd^{-}CD4^{-}CD8^{-}NK1.1^{-}CD11b^{-}CD11c^{-}Ly6C^{+}$
Figure 10K	$CD11c^{-}CD11b^{-}Ly6C^{+}MHCII^{+}$	$CD45^{+}Ly6G^{-}CD19^{-}CD20^{-}CD3^{+/-}TCRgd^{-}CD4^{-}CD8^{-}NK1.1^{-}CD11b^{-}CD11c^{-}Ly6C^{+}MHCII^{+}$
Figure 10L	$CD11c^{-}CD11b^{-}CD103^{+}$	$CD45^{+}Ly6G^{-}CD19^{-}CD20^{-}CD3^{+/-}TCRgd^{-}CD4^{-}CD8^{-}NK1.1^{-}CD11b^{-}CD11c^{-}CD103^{+}$

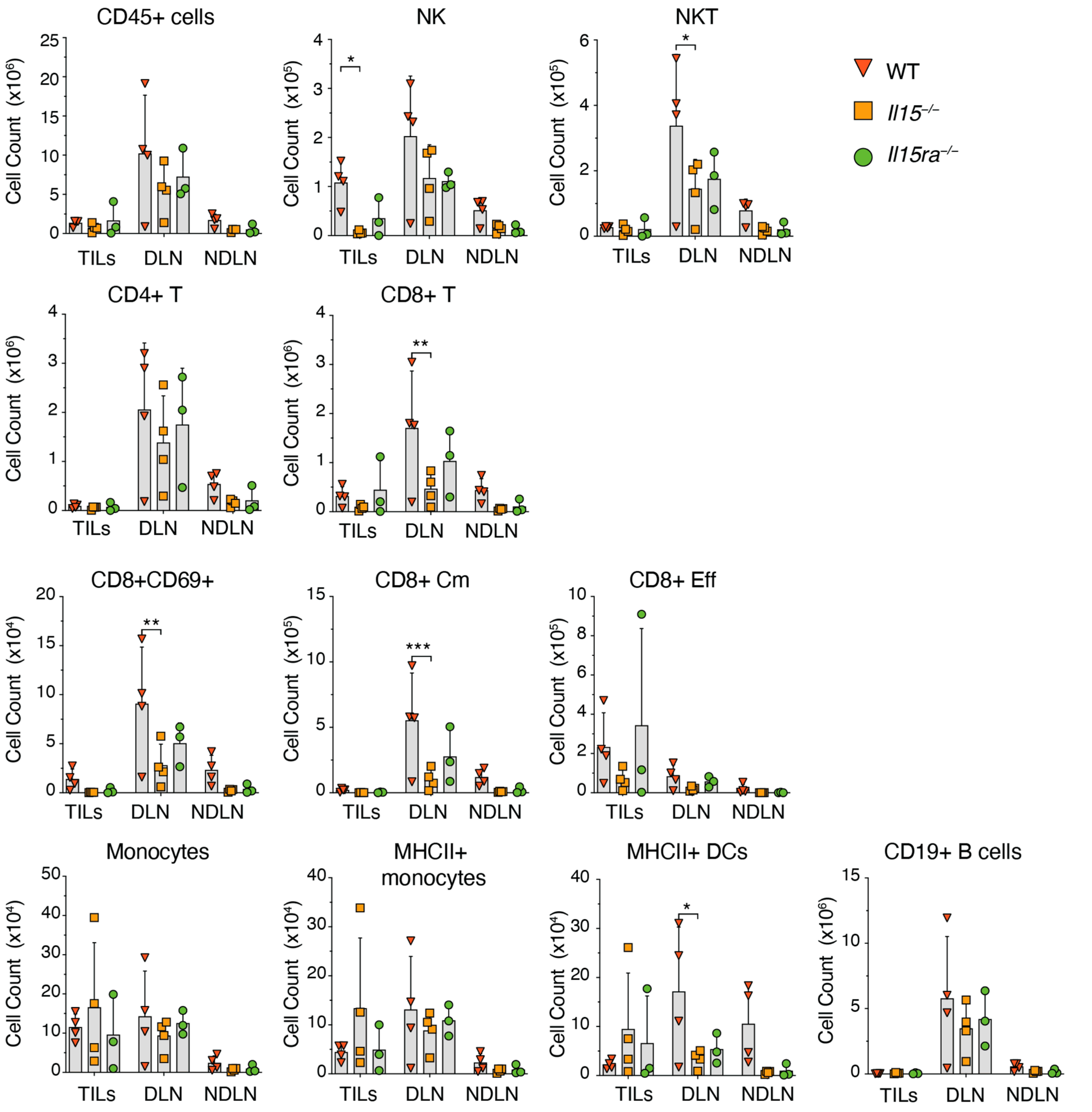

Figure S1. Immune cell infiltration in B16 tumors from WT, *Il15*$^{-/-}$ and *Il15ra*$^{-/-}$ mice. Tumor infiltrating lymphocytes (TILs) and single cell suspensions from tumor draining (DLN) and non-tumor draining (NDLN) lymph nodes were isolated at the time of tumor collection from WT, *Il15*$^{-/-}$ and *Il15ra*$^{-/-}$ mice hosts implanted with B16-F10 melanoma cells. Cells were counted and stained with fluorochrome-conjugated antibodies for lymphoid and myeloid cell markers and analyzed by flow cytometry. Data from 4 mice per group from two independent experiments are shown. The cell numbers in TILs were equalized to the weight of the tumor mass (TILs per gram; TILS(g^{-1})). Absolute number of the indoicated immune cell populations [Leukocytes (CD45$^+$), NK cells (NK1.1$^+$TCRβ$^-$), NKT cells (NK1.1$^+$TCRβ $^+$) cells, CD4$^+$ T cells CD8$^+$ T cells, CD8+ T cell subsets (CD8$^+$ Cm– CD8$^+$CD44$^+$CD62L$^+$ and CD8$^+$ Eff – CD8$^+$CD44$^+$CD62L$^-$), monocytes (CD11b$^+$Ly6C$^+$), DCs (CD11b$^-$CD11c$^+$) and B cells (CD19$^+$)]

were calculated from their percentages within the gated populations. Statistics: Mean + SD. Two-way ANOVA with Tukey's multiple comparison test. * $p \leq 0.05$, ** $p \leq 0.01$, *** $p \leq 0.001$. Abbreviations: CM, central memory; EFF effector; DC, dendritic cells.

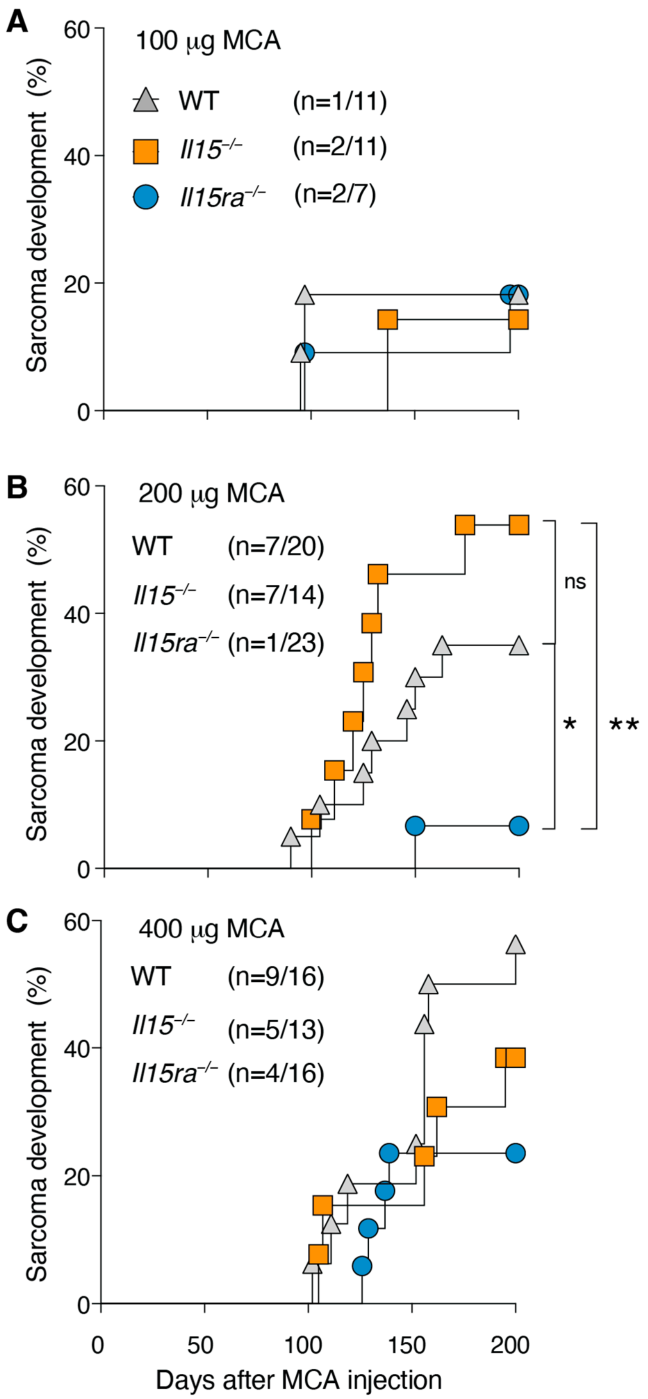

Figure S2. MCA dose-dependent development of fibrosarcomas. WT, *Il15*$^{-/-}$ and *Il15ra*$^{-/-}$ mice were injected the chemical carcinogen 3-ethylcholanthrene (MCA) subcutaneously. Development of fibrosarcoma was monitored visually and by using a digital vernier caliper until the endpoint (tumor diameter of 20mm in any direction, or ulceration at any diameter),

when the animals were euthanized. Kaplan-Meyer curve for the number of mice that developed tumors at 100 (A), 200 (B), 400 μg (C) of MCA.

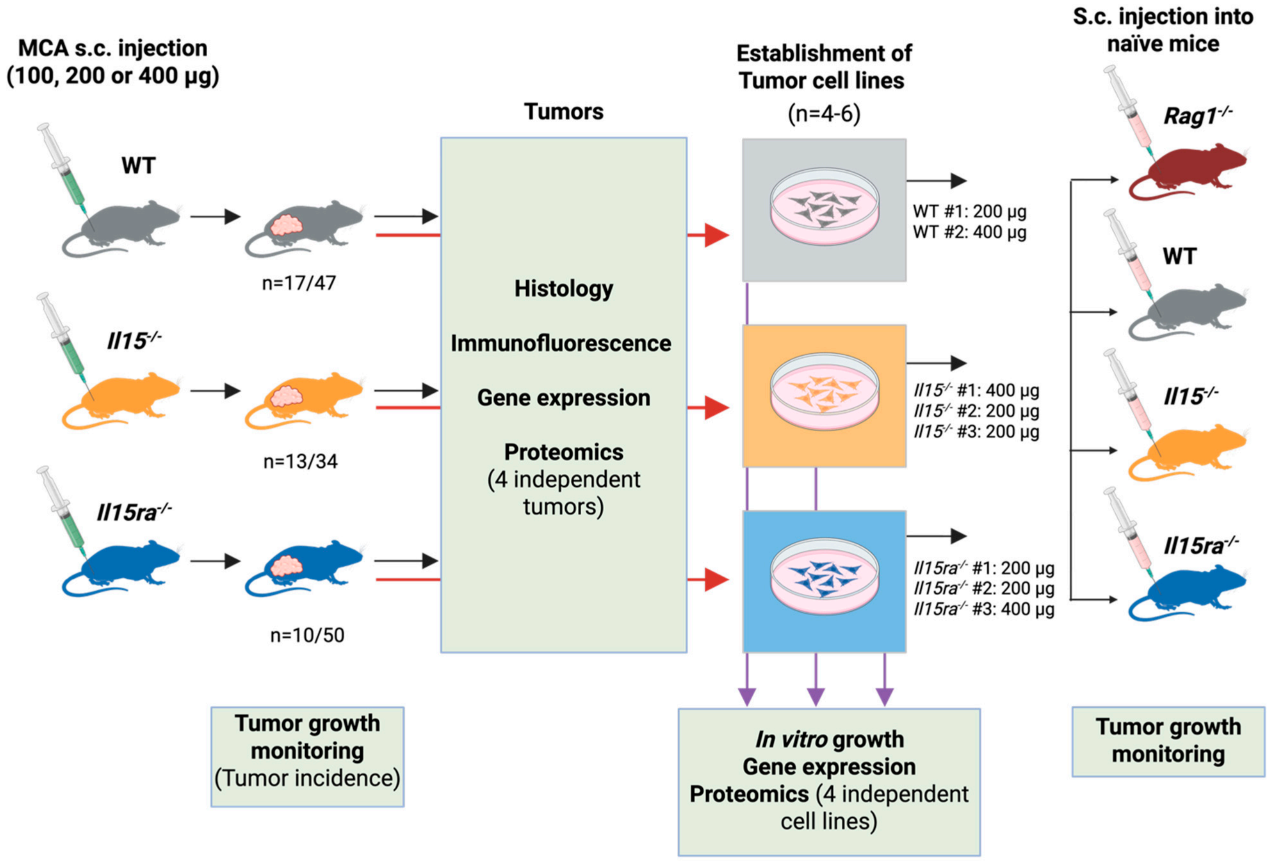

Figure S3. Experimental flow for testing the immunoedited status of MCA-induced tumors.

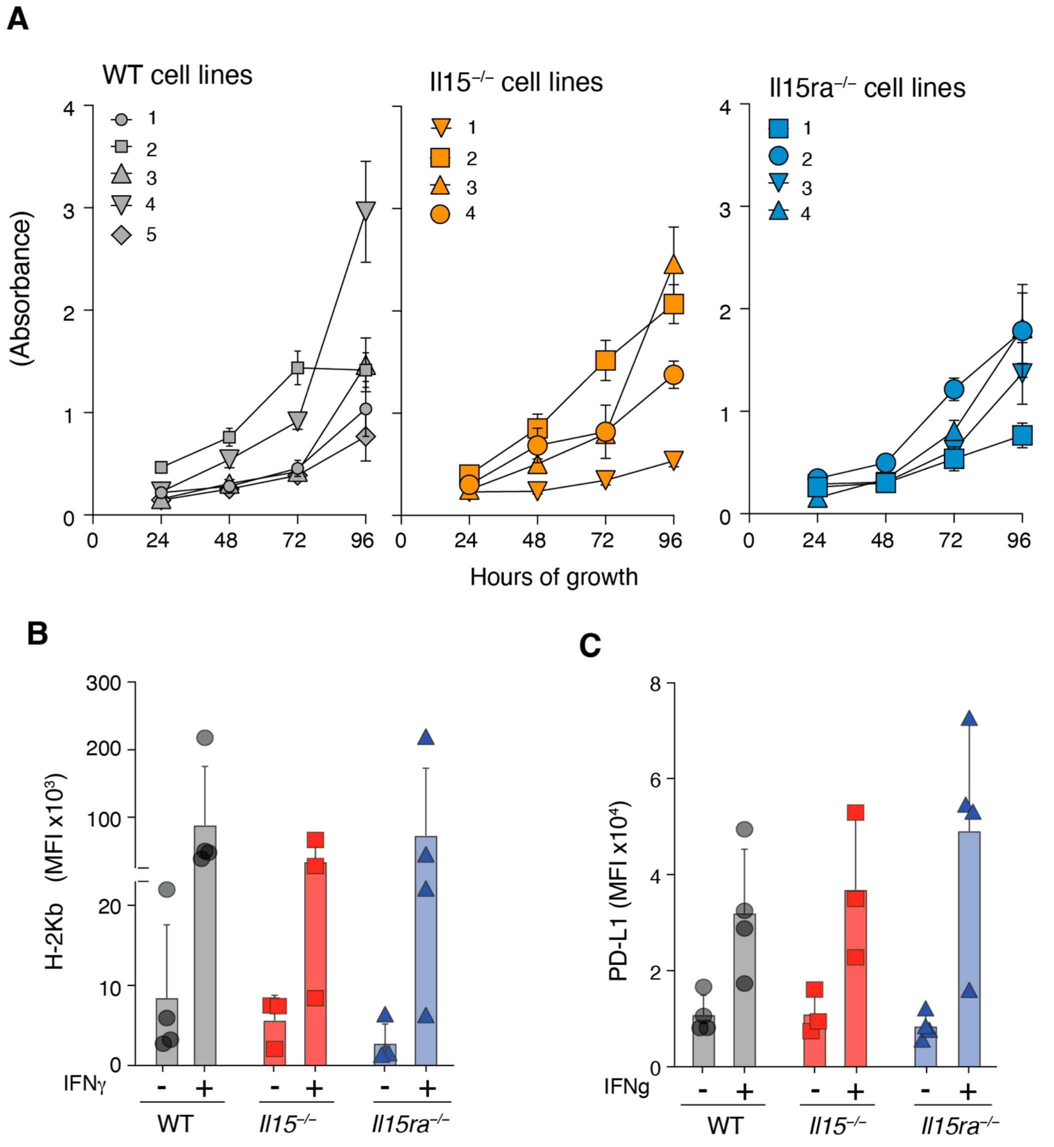

Figure S4. Comparable growth of MCA tumor-derived cell lines *in vitro*. (A) 4-5 independent cell lines were established from the tumors *in vitro*. Growth of the different MCA-tumor derived cell lines *in vitro* as determined by MTT assay. (B,C) Cell lines were exposed to mouse IFNγ (4ng/ml) for 18 h) and the induction of MHC-I (*H2kb*) and PD-L1 (*Cd274*) was evaluated by RT-qPCR.

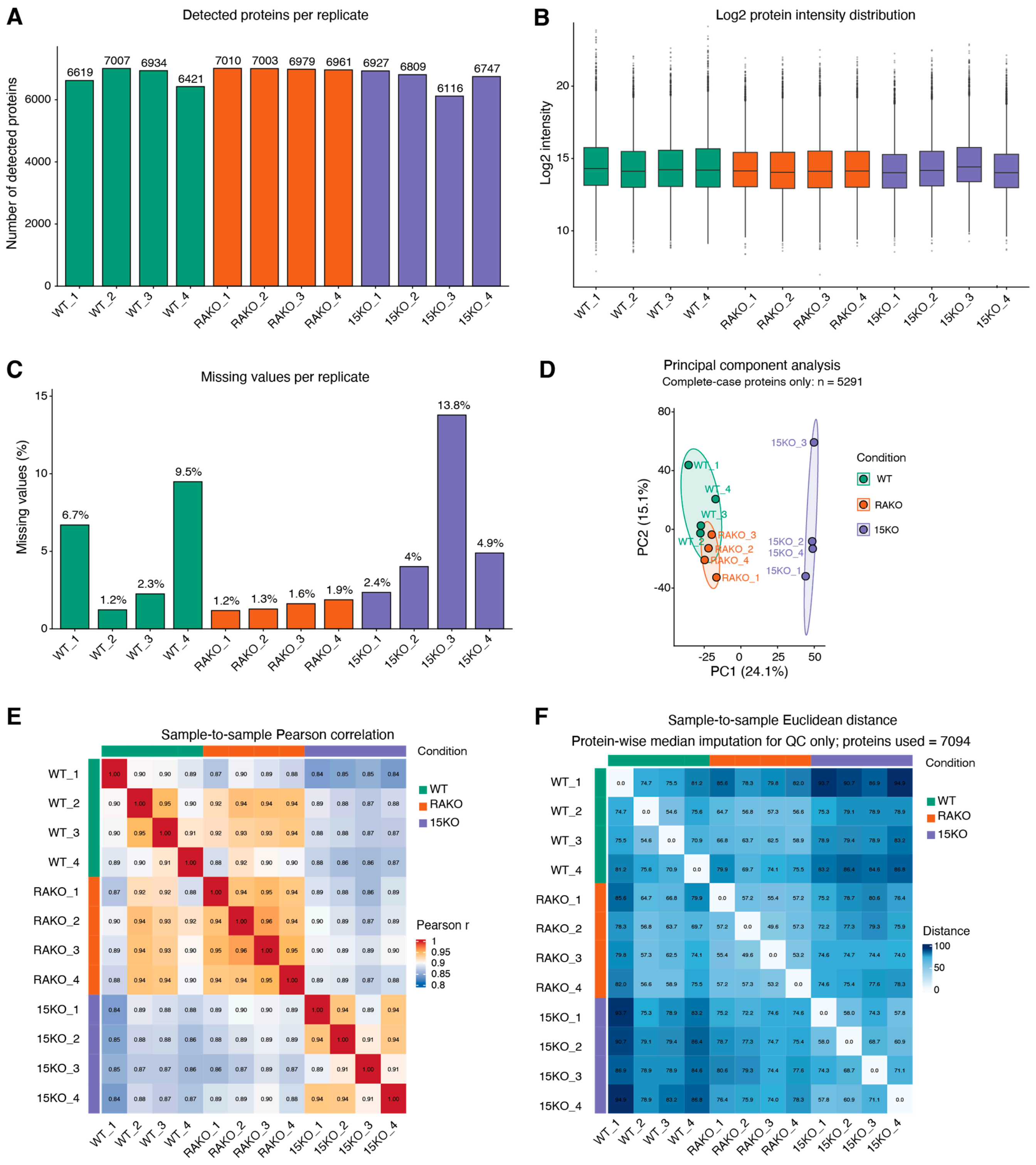

Figure S5. Quality control of mass spectrometry data on MCA tumor proteomes. Quadruplicate samples of proteins extracted from WT, *Il15*$^{-/-}$ (15KO) and *Il15ra*$^{-/-}$ (RAKO) tumors were subjected to shotgun proteomics using LC-MS/MS. (A) Number of individual proteins identified in each tumor by more than one peptide; (B) protein intensity distribution in the samples; (C) Missing value patterns across samples; (D) Principal component analysis (PCA) plot; (E) Pearson correlation between samples; (F) Sample to sample Euclidean distance.

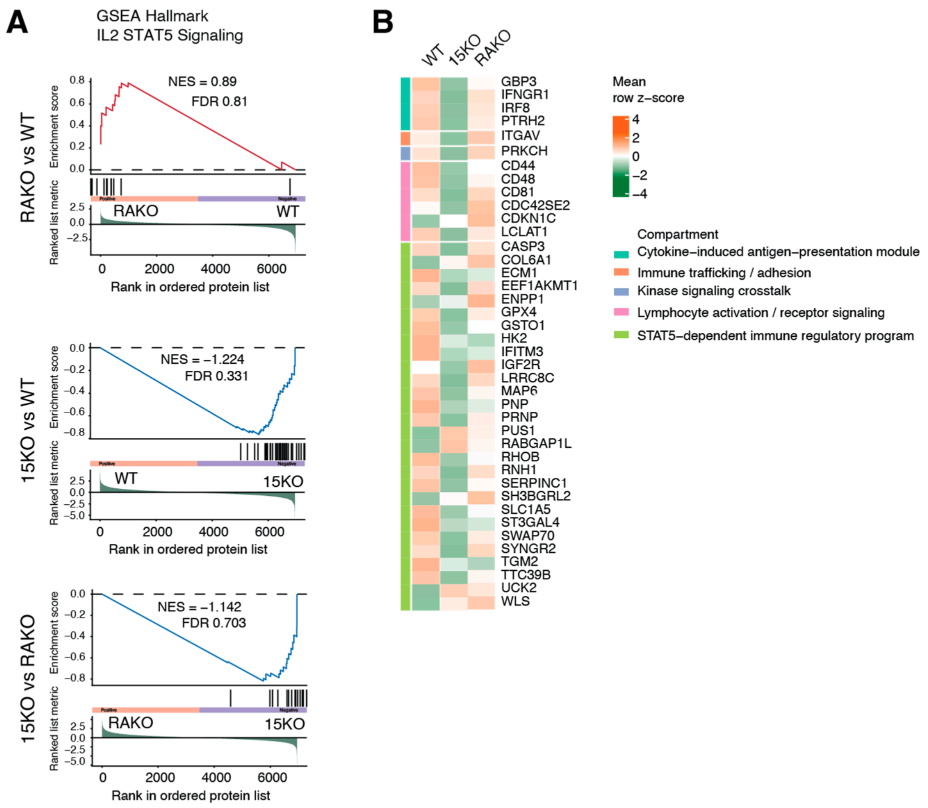

Figure S6. Differential enrichment of IL-2-Stat5 signaling pathway proteins in WT, *Il15ra*$^{-/-}$ and *Il15*$^{-/-}$ tumors. (A) Unbiased GSEA of Hallmark IL-2 Stat5 signaling pathway proteins that are differentially expressed in WT, *Il15ra*$^{-/-}$ and *Il15*$^{-/-}$ tumors. (B) Heatmap representation of normalized abundance levels of the indicated proteins (row Z-score, color coded) within the functional components of IL-2 Stat5 signaling pathway (color coded and indicated) in quadruplicate samples of WT, *Il15ra*$^{-/-}$ and *Il15*$^{-/-}$ tumors.

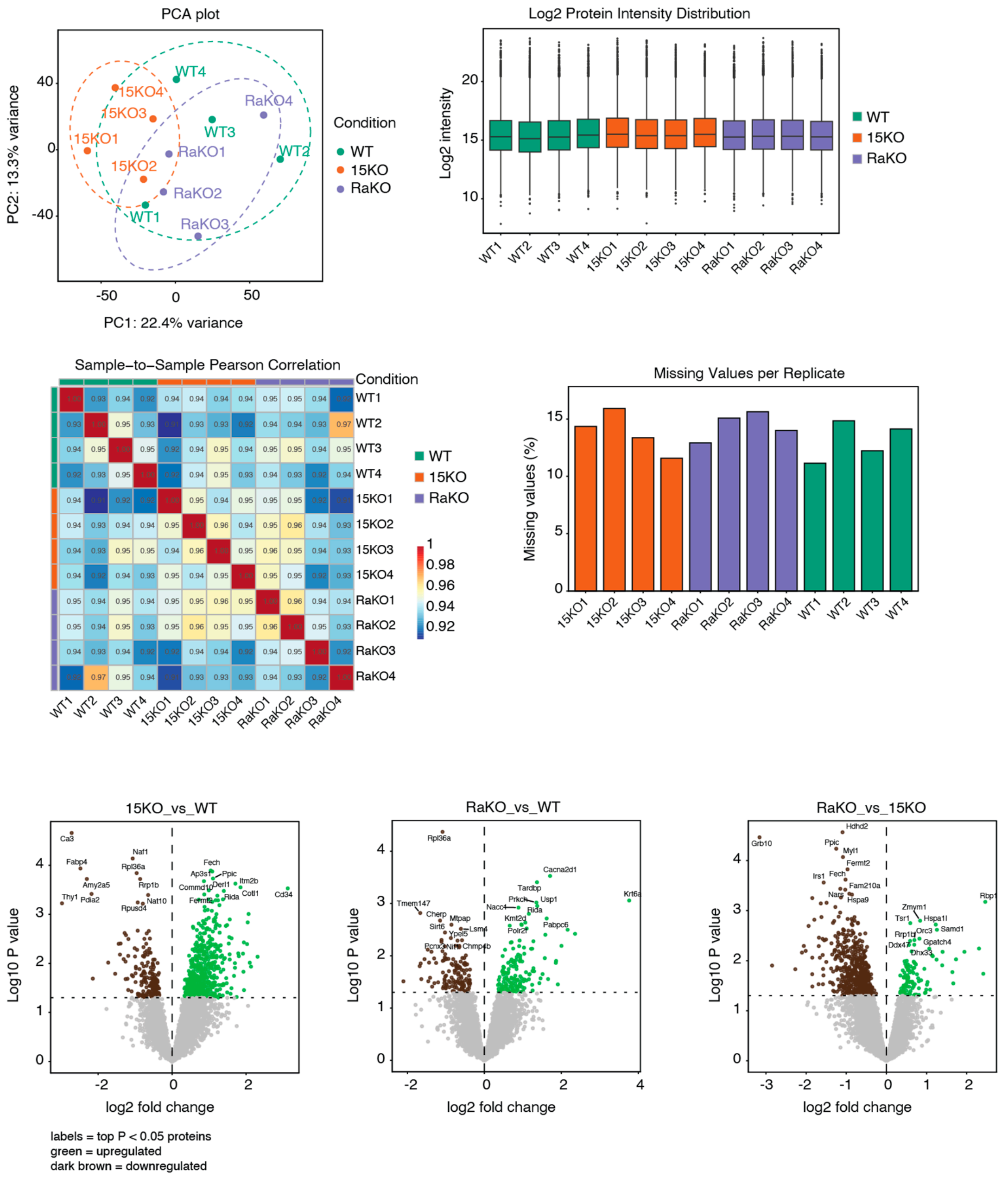

Figure S7. Quality control of mass spectrometry data on proteomes of MCA tumor derived cell lines. Quadruplicate samples of proteins extracted from WT, $Il15ra^{-/-}$ and $Il15^{-/-}$ MCA tumor-derived cell lines were subjected to LC-MS/MS. Principal component analysis (PCA) plot, protein intensity distribution in the samples, Pearson correlation between samples, missing value patterns across samples and volcano plot of proteins detected in IL-15KO and IL-15RαKO compared to WT cell lines, and IL-15KO compared to IL-15RαKO cell lines are shown.

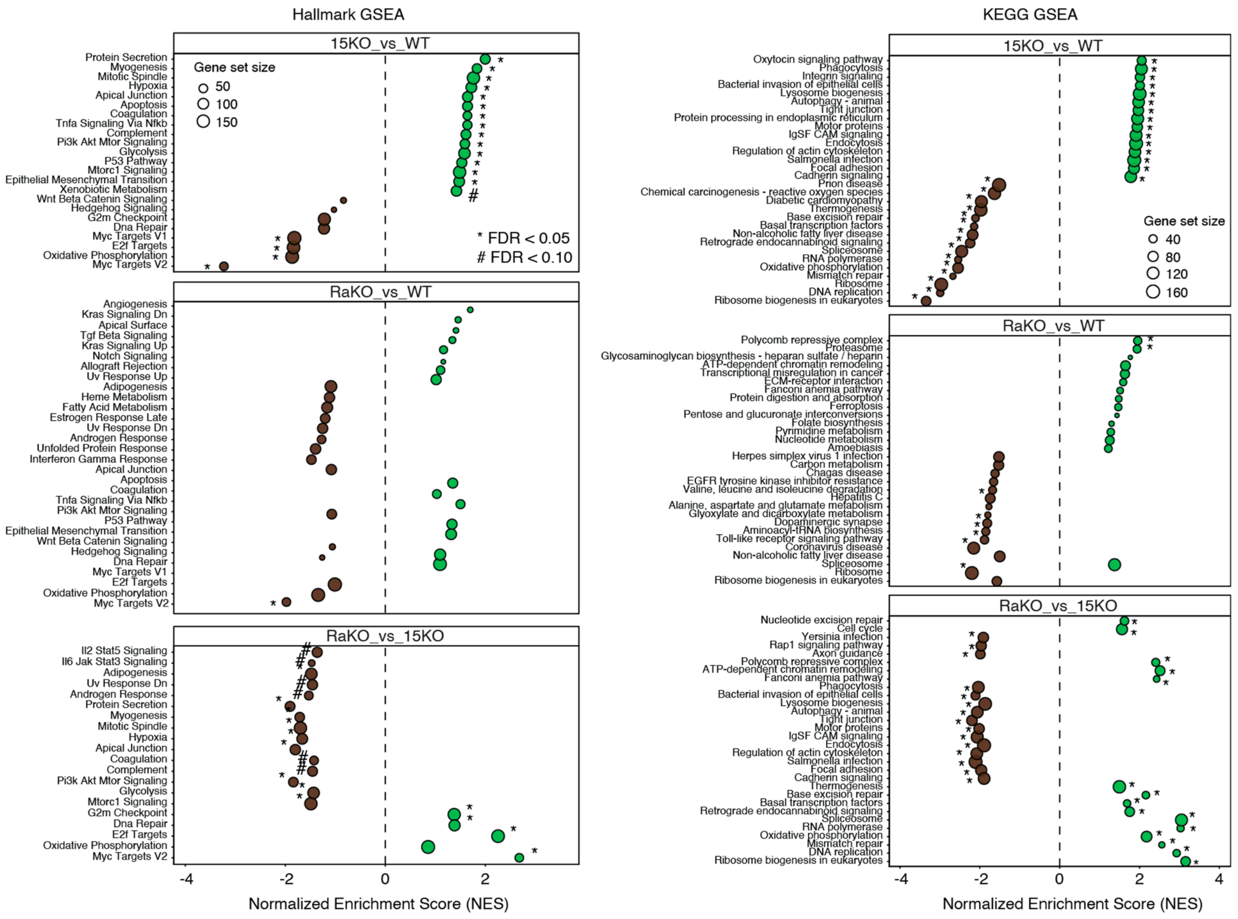

Figure S8. Differentially expressed pathways from proteome analyses of MCA tumor-derived cell lines. Pathways that are different between IL-15KO and IL-15RαKO compared to WT cell lines, and IL-15KO compared to IL-15RαKO cell lines by Hallmark and KEGG GSEA analyses are shown.

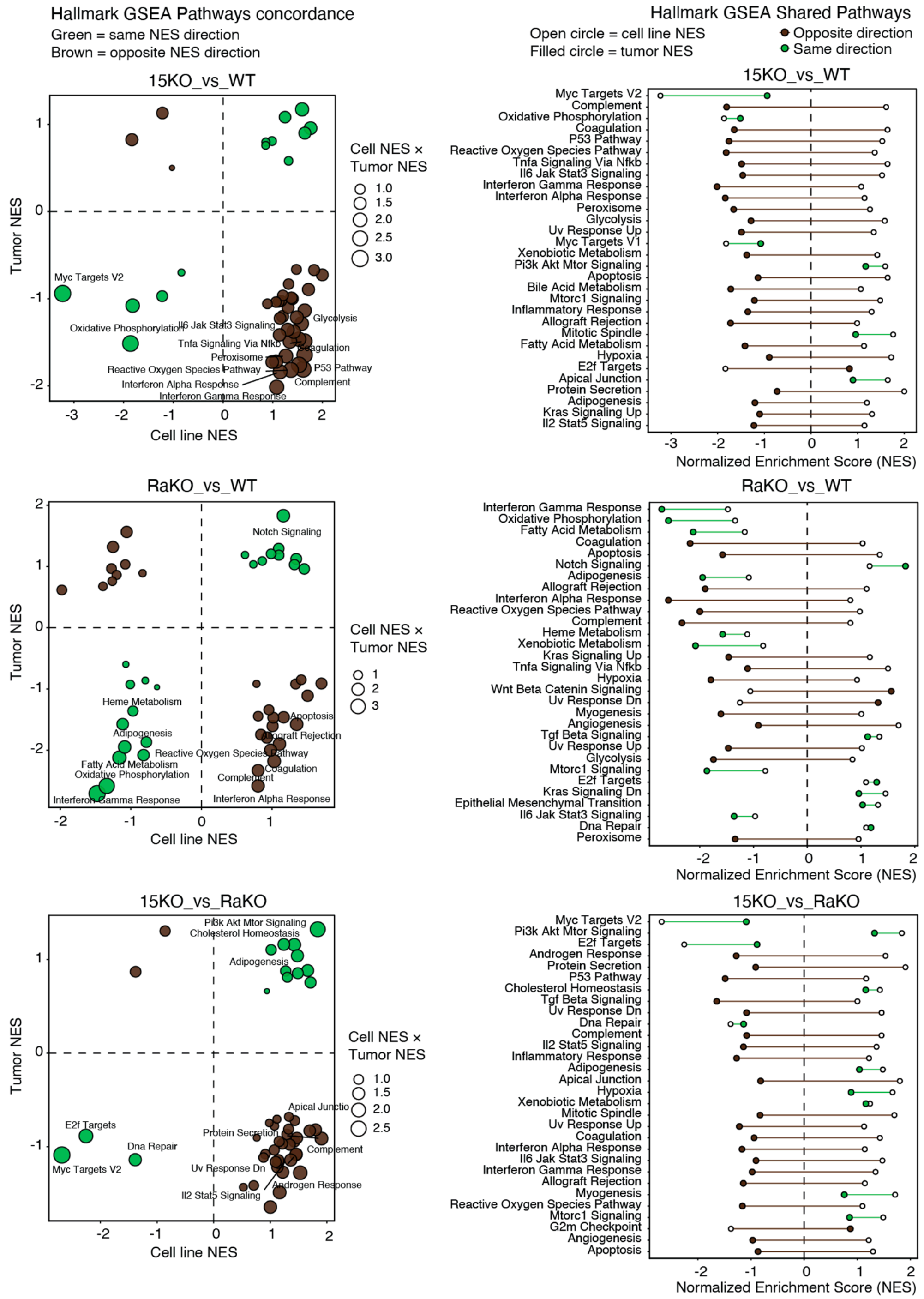

Figure S9. Comparison of differentially expressed pathways from proteome analyses of MCA tumors and MCA tumor derived cell lines. Hallmark Pathway concordance and shared pathways are shown.

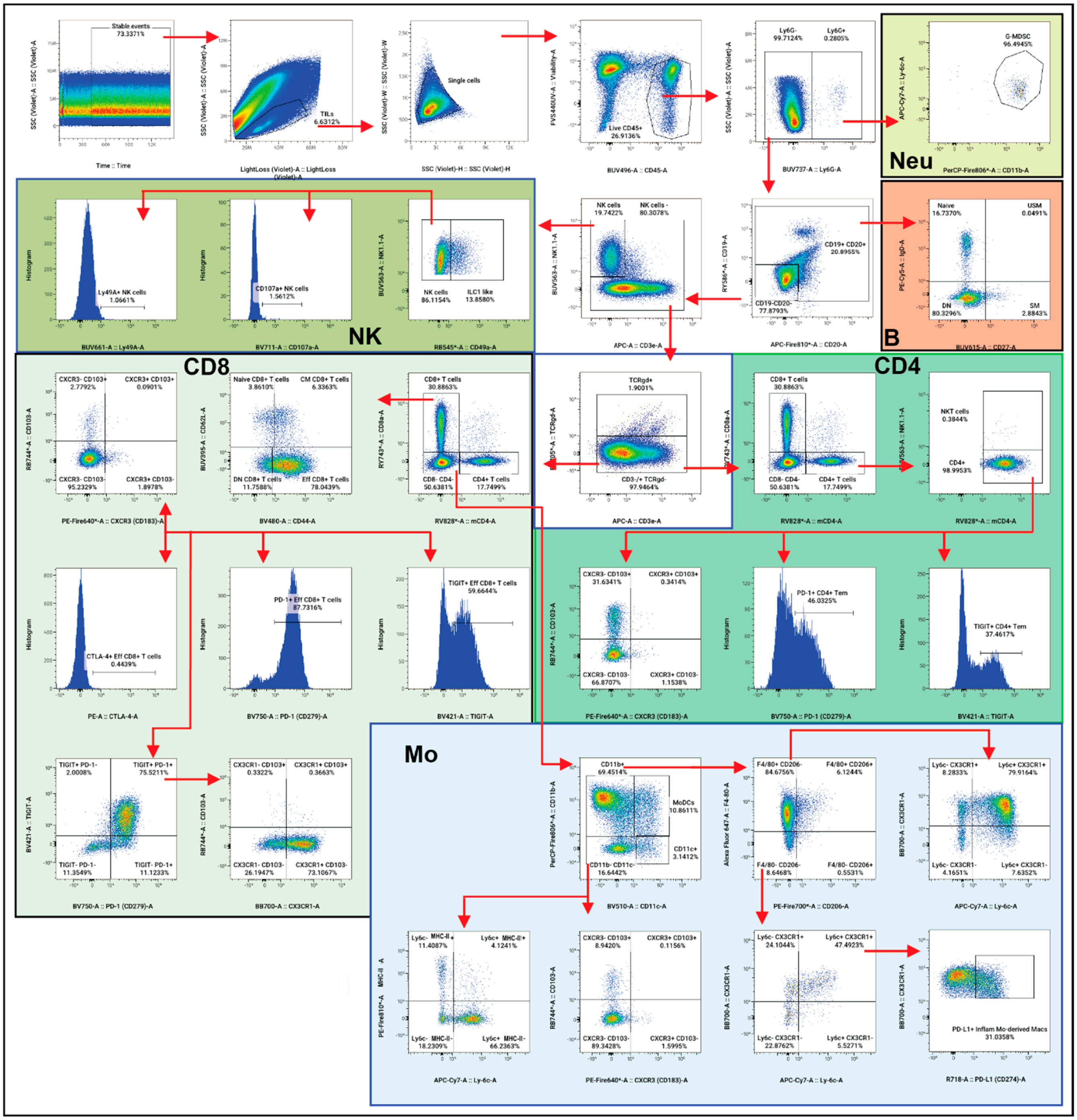

Figure S10. Gating strategy for data shown in Fig. 9 and 10. Arrows indicate the gating sequence. Single cells were gated out for dead cells followed by selection of CD45+ cells. Within CD45+ cells, neutrophils, B cells, NK cells and gd T cells were gated out sequentially. CD4+ and CD8+ T cells were gated out in the remaining population. Monocytes were selected on the CD4-CD8- cells. Abbreviations: Mo, monocyte/macrophage subsets. Neu, neutrophils.

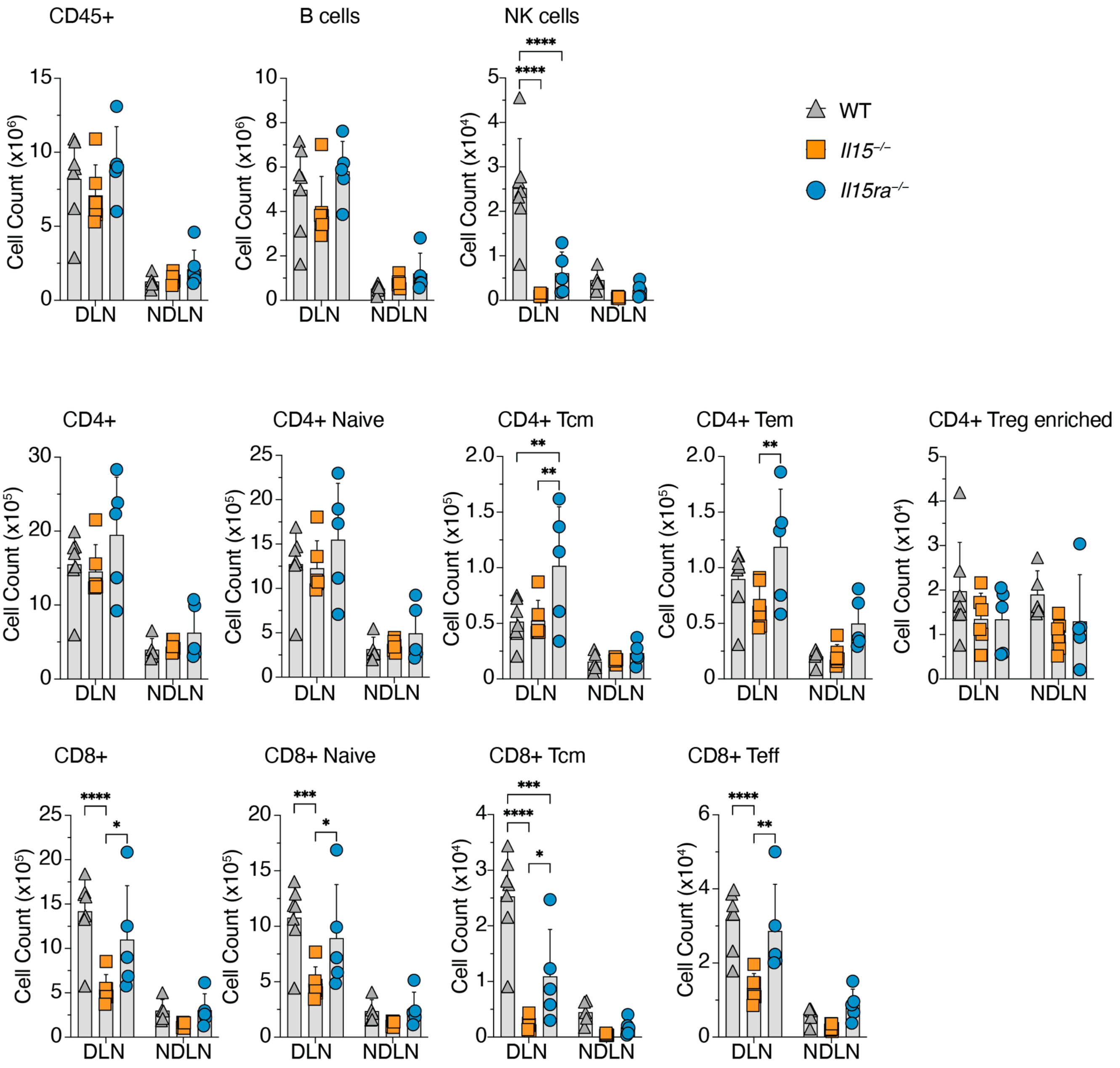

Figure S11. Distribution of select immune subsets detected in the draining and non-draining lymph nodes of B16-NLRC5 tumor bearing mice (data complementary to Figures 8 and 9) Statistics: Mean + SD. Two-way ANOVA with Tukey's multiple comparison test. * $p \leq 0.05$, ** $p \leq 0.01$, *** $p \leq 0.001$. Cm, central memory; Eff, effector T cells.
